# Prospective associations between diet quality, dietary components, and risk of cardiometabolic multimorbidity in older British men

**DOI:** 10.1007/s00394-023-03193-x

**Published:** 2023-06-19

**Authors:** Qiaoye Wang, Amand Floriaan Schmidt, Lucy T. Lennon, Olia Papacosta, Peter H. Whincup, S. Goya Wannamethee

**Affiliations:** 1 Department of Primary Care and Population Health, Institute of Epidemiology and Health Care, University College London, London, NW3 2PF UK; 2 Department of Population Science and Experimental Medicine, Institute of Cardiovascular Science, University College London, London, WC1E 6DD UK; 3 Department of Cardiology, Amsterdam Cardiovascular Sciences, Amsterdam University Medical Centre, University of Amsterdam, Amsterdam, The Netherlands; 4 Population Health Research Institute, St George’s University of London, London, SW17 0RE UK

**Keywords:** Cardiometabolic multimorbidity, Diet, Fish consumption, Prospective cohort study

## Abstract

**Purpose:**

Cardiometabolic multimorbidity (CMM) is a major public health challenge. This study investigated the prospective relationships between diet quality, dietary components, and risk of CMM in older British men.

**Methods:**

We used data from the British Regional Heart Study of 2873 men aged 60–79 free of myocardial infarction (MI), stroke, and type 2 diabetes (T2D) at baseline. CMM was defined as the coexistence of two or more cardiometabolic diseases, including MI, stroke, and T2D. Sourcing baseline food frequency questionnaire, the Elderly Dietary Index (EDI), which was a diet quality score based on Mediterranean diet and MyPyramid for Older Adults, was generated. Cox proportional hazards regression and multi-state model were used to estimate the hazard ratios (HRs) and 95% confidence intervals (CIs).

**Results:**

During a median follow-up of 19.3 years, 891 participants developed first cardiometabolic disease (FCMD), and 109 developed CMM. Cox regression analyses found no significant association between baseline EDI and risk of CMM. However, fish/seafood consumption, a dietary component of the EDI score, was inversely associated with risk of CMM, with HR 0.44 (95% CI 0.26, 0.73) for consuming fish/seafood 1–2 days/week compared to less than 1 day/week after adjustment. Further analyses with multi-state model showed that fish/seafood consumption played a protective role in the transition from FCMD to CMM.

**Conclusions:**

Our study did not find a significant association of baseline EDI with CMM but showed that consuming more fish/seafood per week was associated with a lower risk of transition from FCMD to CMM in older British men.

**Supplementary Information:**

The online version contains supplementary material available at 10.1007/s00394-023-03193-x.

## Introduction

Cardiometabolic multimorbidity (CMM) is a common pattern of multimorbidity, defined as the co-occurrence of two or more cardiometabolic diseases (CMDs), including myocardial infarction (MI), stroke, and type 2 diabetes (T2D). The prevalence of CMM increases with age, and with the population ageing worldwide, CMM is becoming an increasing global health concern [1–4]. Each additional cardiometabolic condition present would increase healthcare costs, complicate medical treatment, impair quality of life, and double the risk of death [1, 5, 6]. Di Angelantonio et al. estimated that a 2-disease CMM would reduce life expectancy by 12 years at the age of 60 years, and a 3-disease CMM was associated with a 15 years of reduced life expectancy, comparing to people without any cardiometabolic disease [1]. In addition, the development of one cardiometabolic condition can substantially increase the risk of developing another. For example, adults with diabetes are nearly twice as likely to have heart disease or stroke as adults without diabetes [7].

There has been well-established evidence of associations between diet quality, dietary components, and risk of single cardiometabolic diseases. Meta-analyses pooling risk estimates of odds ratio (OR), risk ratio (RR), and hazard ratio (HR) from cohort studies showed that high diet quality, as assessed by Mediterranean diet scores (MDS), was inversely related to the risk of acute MI, unspecified stroke, and T2D [relative risks (RRs) and 95% confidence intervals (95% CI) comparing highest versus lowest category of adherence to the Mediterranean diet were 0.70 (0.62, 0.80), 0.73 (0.59, 0.91), 0.79 (0.72, 0.88) for acute MI, unspecified stroke, and T2D, respectively] [8, 9]. Meanwhile, based on meta-analyses of prospective studies focusing on dietary components, higher intakes of vegetables, fruits, and fish were inversely associated with coronary heart disease and stroke [10], while whole grains, fruits, and dairy were associated with reduced risk of type 2 diabetes [11].

While previous studies have carefully evaluated the association between diet and incidence of individual cardiometabolic diseases, very few have considered associations with incidence CMM [3, 4, 12, 13]. Two cross-sectional studies investigating the relationship between diet and CMM risk yielded inconsistent results. Jeong et al. showed that intakes of calcium, potassium, and fruits were inversely associated with the prevalence of CMM pattern [13], but Sakakibara et al. found no significant differences in the daily consumption of fruits and vegetables among CMM groups [4]. In the two prospective studies that examined the associations of diet and development of CMM, although both studies observed significant associations between diet and risk of the first cardiometabolic disease (FCMD), no significance was found between diet and the development of CMM [3, 12]. These two prior prospective studies, however, derived their dietary measures only based on the daily consumptions of a few selected food groups, which were crude and could not reflect participants’ composite diet quality, adherence to established dietary patterns, or detailed dietary intakes. Hence, the prospective relationships between a priori diet quality scores, dietary components, and risk of CMM have yet to be investigated.

Mediterranean-style diet is characterized by higher consumptions of fruits, vegetables, cereals, legumes, and extra-virgin (cold pressed) olive oil, and low-to-moderate intakes of fish, other meat, dairy products, and red wine [14]. Mediterranean diet score (MDS), which reflects adherence to the Mediterranean-style dietary pattern, is an internationally recognized dietary index that has been broadly studied [8, 9]. Biologically, adhering to the Mediterranean dietary pattern collectively was identified with improving risk factors for cardioembolic diseases, including improved blood pressure, blood sugar, and blood lipid profiles [15]. Considering that participants in the current study are all older adults over 60 years, we applied an MDS-adapted diet quality index, Elderly Dietary Index (EDI), to specifically address adherence to nutritional recommendations for older adults [16]. Prior studies have shown that the EDI was associated with cardiovascular disease risk factors in the Mediterranean population and risk of coronary heart disease in the older British men population [16, 17]. Therefore, to extend the current literatures, aim of this study was to examine the prospective association of diet quality score EDI with risk of CMM, in a cohort of older British men. In addition, we investigated each dietary food component of the EDI on their relationships with CMM risk.

## Methods

### Study design

The British Regional Heart Study (BRHS) follows a cohort of 7735 men aged 40–59 years randomly selected from 24 British towns between 1978 and 1980 [18–20]. This analysis uses baseline data collected from 1998 to 2000, marked as the 20 year follow-up (Q20) of BRHS. A total of 4252 men aged 60–79 completed the Q20 follow-up questionnaire on sociodemographic, health, medication, and general lifestyles, together with a food frequency questionnaire (FFQ), and were invited to a physical examination [19, 20]. Cardiometabolic disease events and deaths were followed till June 2018. We excluded participants with a history of myocardial infarction, stroke, and those who had a doctor-diagnosed or screen-detected type 2 diabetes (fasting plasma glucose $\ge$ 7 mmol/L) at baseline (*n* = 1085). Participants with any missing dietary or demographic data were also excluded (*n* = 294), leaving 2873 men in the present analyses. All participants provided informed written consent in accordance with the Declaration of Helsinki. Ethical approval was obtained from The National Research Ethics Service (NRES) Committee London–Central (Reference number: MREC/02/2/91).

### Assessment of diet quality

Dietary information was obtained through the baseline self-administered FFQ. The FFQ was developed for use in the WHO’s Monitoring Trends and Determinants in Cardiovascular Diseases [21] and has been validated against weighted food consumption in British populations [22, 23]. Participants were asked to report their consumption frequency of 86 different types of food items. For each food item, respondents selected their usual intake frequency from nine options: 1, 2, 3, 4, 5, 6, or 7 days/week; monthly; or rarely/never. In addition, information on specific types of fruits, milk, bread, fat used for cooking and spreading, and household consumption amount of fat and oils were collected in the FFQ.

**Fig. 1:**
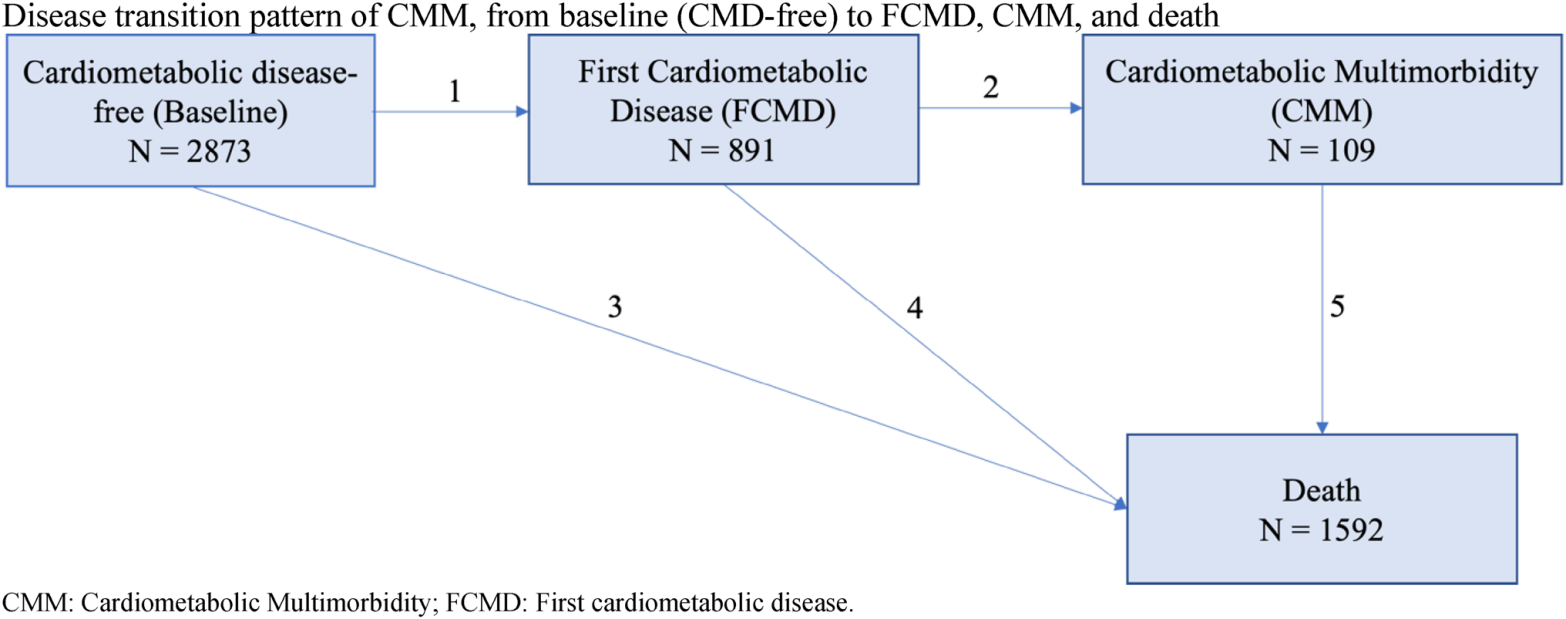
Disease transition pattern of CMM, from baseline (CMD-free) to FCMD, CMM, and death

The EDI was computed from the FFQ. Developed by Kourlaba et al. [16] according to select features of the traditional Mediterranean diet and Modified MyPyramid for Older Adults, the original EDI consists of ten dietary food groups: fruits, vegetables, cereals, legumes, meat, fish and seafood, bread, olive oil, dairy, and alcohol. In the current study, we excluded alcohol from the EDI because of lacking information on the frequency of red wine consumption. Hence, EDI in this study is composed of nine dietary food groups. For each food group, a 4-point scoring system was assigned based on the consumption frequency. A score of 1 means that the consumption frequency adheres the least to the nutritional recommendations, while a score of 4 indicates the healthiest intake frequency of the food group. Since olive oil consumption frequency was unavailable, the scoring of olive oil was modified using never/rarely consumed and tertiles of weekly consumption in the current study. The total EDI score ranges from 9 to 36 (Online Resource Table 1 and Fig. 1), with higher scores indicating better adherence to the dietary recommendations and hence a presumably healthier diet. For the analyses, participants were categorized into quartiles of EDI to reflect their diet quality, using integer cutoffs that produce groups as equally sized as possible (Quartile cutoffs are score 22, 24, and 26).

**Table 1:** Baseline characteristics of 2873 older British men by quartiles of baseline Elderly Dietary Index (EDI) in 1998–2000

Characteristic	EDI quartile 1 (*N* = 793)	EDI quartile 2 (*N* = 749)	EDI quartile 3 (*N* = 651)	EDI quartile 4 (*N* = 680)	*P* value
Age at baseline, years	68.5 (5.5)	68.3 (5.5)	68.2 (5.7)	68.0 (5.3)	0.3
Incident CMM, %	4.2	3.3	4.0	3.7	0.8
Current/recent smokers, %	30.1	14.3	10.0	5.2	< 0.01
Heavy alcohol drinkers, %	4.2	2.8	2.9	1.4	< 0.01
Physically inactive, %	12.7	8.0	7.4	6.4	< 0.01
Manual social class, %	57.4	52.2	37.7	31.3	< 0.01
National IMD most deprived quintile, %	21.6	16.8	11.1	8.1	< 0.01
BMI, kg/m^2^	26.8 (4.0)	26.8 (3.6)	26.8 (3.3)	26.4 (3.2)	0.01
Waist circumference, cm	97.1 (10.9)	96.7 (10.5)	96.5 (9.2)	95.2 (9.2)	< 0.01
Energy intake, kcal/day	2168.2 (571.2)	2189.6 (534.6)	2172.2 (522.6)	2059.8 (440.5)	< 0.01
Family history of diabetes, %	10.3	10.5	11.8	11.7	0.9
Atrial fibrillation based on ECG, %	2.7	3.4	2.7	2.6	0.8
Use of any lipid-lowering drugs, %	2.9	2.9	2.9	5.6	0.01
Use of any blood pressure lowering drugs, %	22.6	24.8	24.2	25.5	0.5
Systolic blood pressure, mm Hg	149.3 (24.2)	149.4 (23.4)	149.8 (23.2)	148.6 (23.7)	0.8
Diastolic blood pressure, mmHg	85.6 (10.9)	85.8 (11.0)	85.8 (10.7)	85.8 (10.7)	0.8
Total cholesterol, mmol/L	6.1 (1.1)	6.0 (1.1)	6.1 (1.0)	6.1 (1.0)	0.3
Plasma HDL cholesterol, mmol/L	1.3 (0.4)	1.3 (0.3)	1.4 (0.3)	1.4 (0.3)	0.06
Plasma LDL cholesterol, mmol/L	4.0 (1.0)	4.0 (1.0)	4.0 (0.9)	4.0 (0.9)	0.3
Triglycerides, mmol/L	1.9 (1.2)	1.8 (1.0)	1.7 (0.8)	1.7 (0.9)	0.5

Values are presented as Mean (SD) or percentage unless stated otherwise

Pearson’s chi-squared test was used for all categorical variables

Kruskal-Wallis test was used for all continuous variables

*CMM* cardiometabolic multimorbidity, *BMI* body mass index, *IMD* Index of multiple deprivation

### Ascertainment of cardiometabolic disease and cardiometabolic multimorbidity

Participants were followed up for MI, stroke, T2D, and death to June 1st, 2018. Both nonfatal and fatal MI and stroke were counted as diagnoses of cardiometabolic diseases. Nonfatal cardiometabolic diseases were ascertained from ongoing general practitioner reports and reviews of participants’ medical records biennially [20]. Fatal MI and stroke events were determined through the death information that was obtained from the National Health Service Central Registers in Southport (for England and Wales) and Edinburgh (for Scotland). Causes of death were coded using the *International Classification of Diseases, Ninth Revision*. Fatal MI was coded as 410–414, and fatal stroke was coded as 430–438. CMM was ascertained when the participant was diagnosed or died of a second kind of cardiometabolic disease.

### Available covariates

Smoking status, alcohol intake, physical activity, social class, current use of any lipid-lowering drugs, and energy intake were obtained from the baseline self-completed questionnaire [24–26]. Height, weight, and waist circumference were measured at the baseline physical examination, as described previously [27]. BMI was calculated using height and weight measurements. The National Index of Multiple Deprivation (IMD) was computed from the baseline neighborhood-level socioeconomic factors, including income, employment, education, housing, and living environment, and were divided into quintiles from the least to the most deprived [28]. Participants were classified into four smoking groups: never, long-term ex-smokers (gave up > 15 years), ex-smokers (gave up between 5 and 15 years), or current/recent smokers (gave up $\le$ 5 years). Alcohol intake was categorized into six groups on the basis of the number and frequency of alcoholic beverage consumption: none, occasional, light, moderate, heavy, or unspecified for those who did not provide information on the amount they drank. Based on the self-reported exercise frequency and intensity, physical activity was classified into six groups: inactive, occasional, light, moderate, moderately vigorous/vigorous, or unspecified for men lacking the information. Social class was categorized into manual, non-manual, or unspecified based on the longest held occupation. Current use of any lipid-lowering drugs was derived by self-reported information on the use of statin or other lipid lowering drugs. Energy intake was calculated by multiplying energy values (kcals) with macronutrient intakes, which were computed by a validated computer program that multiplied participants’ food consumption frequency by each food’s standard portion size and by the food’s nutrient compositions obtained from the UK food-composition tables [29, 30].

### Statistical methods

Participants who were cardiometabolic disease-free at baseline were grouped by their baseline EDI quartiles. For the four groups, baseline characteristics were summarized as means with standard deviation for continuous variables and percentage for categorical variables. Kruskal-Wallis test was used to compare the difference of continuous variables, and Pearson’s chi-squared test was conducted to compare the difference of categorical variables.

Cox proportional hazards models were first carried out to estimate the associations of the baseline EDI and its dietary food components with risk of CMM. Each participant’s follow-up time was computed from the date of the Q20 physical examination to the date of event, death, or end of follow-up (June 1st, 2018), whichever occurred first. Specifically, CMM event date represented the date when the participant was diagnosed or died of a second kind of cardiometabolic disease during follow-up. Age was adjusted in model 1. Model 2 made extra adjustment with BMI. And model 3 included other sociodemographic and lifestyle variables, including waist circumference, smoking, alcohol intake, physical activity, social class, National IMD, use of any lipid-lowering drugs, and energy intake. Patients with heart failure were found to have higher risk of developing cardiometabolic diseases and may have poorer or better dietary habit compared to people without a history of heart failure [31–34]. We therefore conducted a sensitivity analysis by excluding participants with doctor-diagnosed heart failure at baseline to test the robustness of our analyses. In addition, to investigate the potential impact of missing observations, we carried out missing data imputation. Missing variables were imputed through multiple imputation using chained equations (*n* = 10). All cox models were examined for the proportional-hazards assumption using the Kolmogorov-type supremum test and found no trend with time. *P* values for linear trend were computed and presented for the test of associations between baseline EDI quartiles, groups of EDI food components, and risk of CMM. In addition, a *P* value < 0.05 was considered statistically significant.

After Cox regression analyses, we further conducted multi-state models to assess associations of the baseline EDI score quartiles and significant food components of the EDI with the disease transitions from baseline (free of CMD) to FCMD, and to CMM [35]. Death was included in the transition pattern as the absorbing state. Therefore, there were five transition stages in the CMM disease transition pattern without differentiating FCMD: (1) Baseline (free of CMD) to FCMD; (2) FCMD to CMM; (3) Baseline to death; (4) FCMD to death; and (5) CMM to death. We further divided FCMD into individual diseases (i.e., first MI, first stroke, and first T2D) and there were eleven transition stages in the CMM disease transition pattern (Online Resource Fig. 2). For participants entered different states on the same date (*n* = 321), we added 0.5 days to the entering date of the latter state to maximize the numbers being used in the model. For example, for participants who had FCMD and death at the same date, the date of death equals to the date of FCMD plus 0.5 day. After obtaining multi-state model estimates, we then predicted the probabilities of being an FCMD-survivor (developed FCMD without transitioning to another state), CMM-survivor (developed CMM without transitioning to another state), dead without FCMD, dead with FCMD, and dead with CMM from the enrollment to the longest follow-up. All covariates were set to average or reference levels of the BRHS population in the present analyses. Probabilities were obtained separately for participants in each baseline EDI quartiles and each consumption frequency groups of the EDI food components. Analyses were conducted using R (version 4.0.3) and the multi-state model was performed with “mstate” package.

## Results

### Baseline characteristics of study participants

Of 2873 participants included in the current analyses, after a median follow-up of 19.3 years (Q1, Q3: 18.8, 19.9), 891 participants developed FCMD, 109 people proceeded to develop CMM, and 1592 participants died with or without developing FCMD (Fig. 1). Median time for development of FCMD was 8.3 years (Q1, Q3: 4.5, 13.1), and median time for CMM development was 13.8 years (Q1, Q3: 9.1, 16.8). Of 891 cases of FCMD, 354 of them were MI, 285 were stroke, and 252 were T2D (Online Resource Fig. 2). About half of the people who experienced a first event of MI or stroke, experienced a fatal event.

Table 1 presents the baseline characteristics of BRHS participants by their EDI quartiles in 1998–2000. Participants who had closer adherence to the Mediterranean diet (measured by EDI) at baseline were less likely to be current/recent smokers, heavy alcohol drinkers, manual workers, being physically inactive, or being in the most deprived quintile of the National IMD. Also, participants in the highest quartile of EDI had significantly smaller BMI, waist circumference, and total energy intake at baseline. Participants with missing data tend to be older and had less energy intakes, also were more likely to be current/recent smokers or ex-smokers, heavy drinkers, manual workers, being physically inactive, and having lower plasma total cholesterol and lower plasma LDL-C (Online Resource Table 2).

### Diet and incident cardiometabolic multimorbidity

Cox regression analyses showed that baseline EDI were not significantly associated with risk of CMM after adjusting for sociodemographic and lifestyle factors (Table 2). No consistent trend was found between quartiles of baseline EDI and CMM risk, with being in Q2 having the lowest estimated risk compared to participants in Q1 in the fully adjusted model (HR: 0.74, 95% CI 0.44, 1.26).

**Table 2:** Prospective association between baseline Elderly Dietary Index and incident cardiometabolic multimorbidity in BRHS participants aged 60–79 years in 1998–2000 (*n* = 2873)

Baseline EDI	No. of events	Rate (per 1000 person-years)	HR (95% CI) of CMM		
			Model 1^a^	Model 2^b^	Model 3^c^
Per SD increase			0.85 (0.70, 1.03)	0.87 (0.72, 1.06)	0.93 (0.76, 1.15)
Q1 (EDI Score 9–22, *n* = 793)	33	2.08	Ref	Ref	Ref
Q2 (EDI score 23–24, *n* = 749)	25	1.67	0.67 (0.40, 1.12)	0.69 (0.41, 1.16)	0.74 (0.44, 1.26)
Q3 (EDI score 25–26, *n* = 651)	26	2.00	0.81 (0.49, 1.35)	0.83 (0.50, 1.38)	0.94 (0.55, 1.60)
Q4 (EDI score 27–35, *n* = 680)	25	1.84	0.70 (0.42, 1.17)	0.77 (0.46, 1.28)	0.89 (0.51, 1.54)
*P* for trend			0.26	0.39	0.86

*EDI* Elderly Dietary Index, *CMM* Cardiometabolic multimorbidity, *HR* Hazard ratio, *CI* Confidence interval

^a^Model 1: adjusted for age

^b^Model 2: adjusted for model 1 + BMI

^c^Model 3: adjusted for model 2 + waist circumference, smoking status, alcohol intake, physical activity, social class, National IMD, energy intake, and use of any lipid-lowering drugs

*P < 0.05

**Fig. 2:**
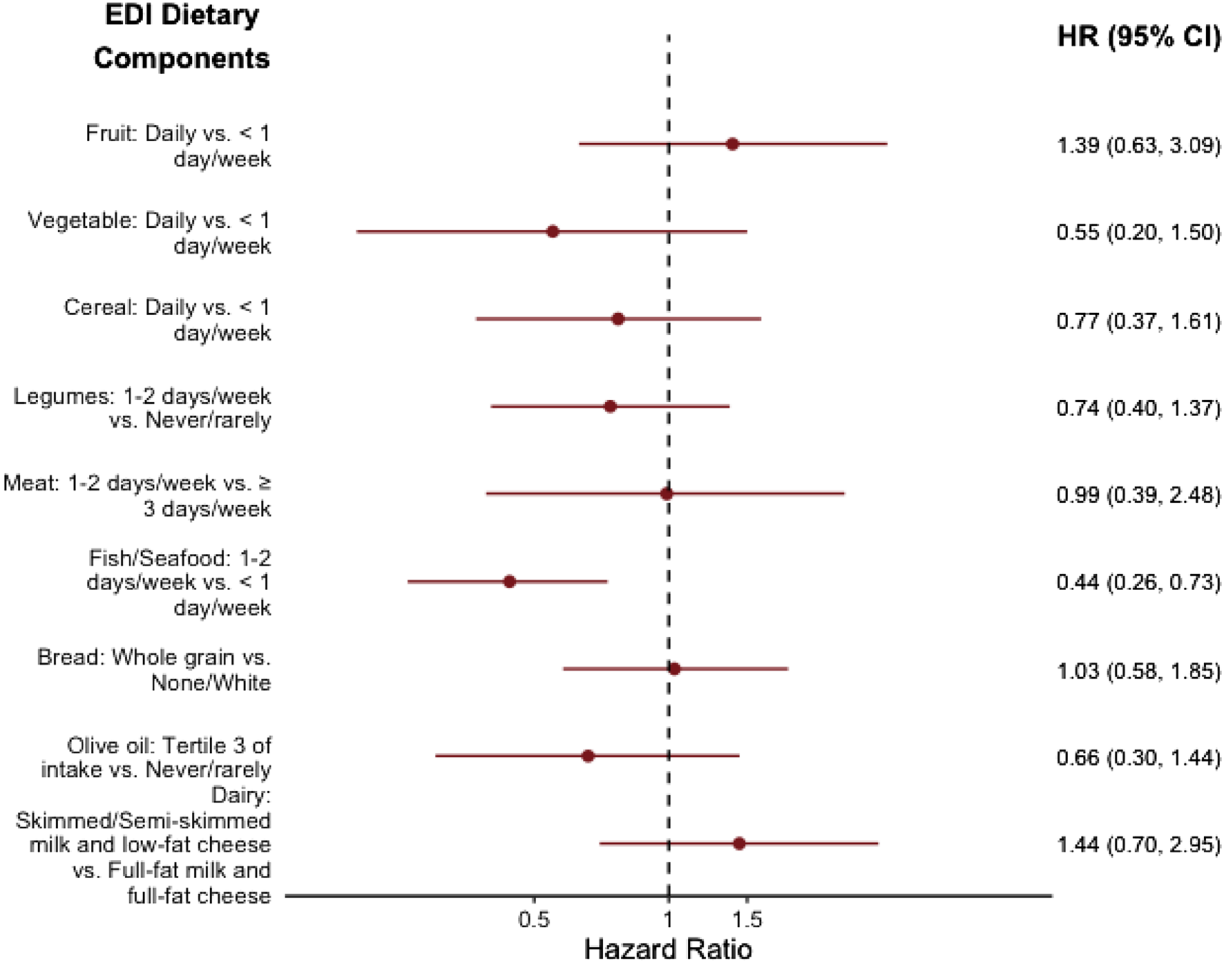
Prospective associations between individual dietary component of the Elderly Dietary Index and incident cardiometabolic multimorbidity in BRHS participants aged 60–79 years in 1998–2000 (*n* = 2873). EDI Elderly Dietary Index, HR Hazard ratio, CI Confidence interval. Multivariable model was adjusted for age, BMI, waist circumference, smoking status, alcohol intake, physical activity, social class, National IMD, energy intake, use of any lipid-lowering drugs, and modified EDI score without food group of interest. Dietary components were derived from the baseline FFQ as categorical variables, which were defined by the EDI criteria. The reference level was chosen to be the least intake adherence to the dietary pattern. For fish/seafood and bread consumptions, the two least adherence intake groups were combined (never/rarely and < 1 day/week, none and white, respectively) due to small case numbers in the least consumption adherence group

For the Cox regression analyses of individual dietary components of the EDI with CMM risk, we found that fish/seafood consumption was significantly associated with risk of CMM (Fig. 2). Consuming the presumably healthiest amount of fish/seafood at baseline, which was 1–2 days/week according to EDI’s scoring criteria, was significantly associated with lower risk of CMM (HR: 0.44, 95% CI 0.26, 0.73), compared to consuming fish/seafood < 1 day/week, after adjusting for sociodemographic and lifestyle covariates. In addition, it was found that eating fish/seafood $\ge$ 3 days/week, defined as the second healthy intake frequency in the EDI fish/seafood group, was also significantly associated with lower CMM risk compared to consumption of < 1 day/week (HR: 0.37, 95% CI 0.20, 0.66, Online Resource Table 3), after adjusting for all other covariates. Excluding men with prevalent heart failure at baseline (*n* = 14) or imputing missing data made little difference to the findings between baseline EDI, fish/seafood consumption, and risk of CMM (Online Resource Tables 4 and 5).

### Diet and cardiometabolic multimorbidity transitions

Table 3 shows the multi-state model results of transition risk estimates from baseline (CMD-free) to FCMD and to CMM by baseline EDI quartiles. Baseline EDI quartiles were not significantly associated with the risk of FCMD, nor with the risk of CMM. Full disease transition results showed that being in higher baseline EDI quartiles was significantly related to lower risk of mortality transitioning from FCMD, particularly from first stroke or first T2D to death (Online Resource Tables 6, 7). However, significant higher risk of CMM transitioning from first MI and risk of death transitioning from CMM were found comparing baseline EDI Q3 to Q1 (Online Resource Table 7). Holding average or reference levels of covariates in the analyses, at 20 year follow-up, the percentages of participants predicted to develop FCMD or CMM did not differ significantly across baseline EDI quartiles (Online Resource Fig. 3). But the predicted risk of death (with or without FCMD and CMM) was approximately 10% higher for participants being in the baseline EDI Q1, comparing to participants being in the other three quartiles (Online Research Fig. 3).

**Table 3:** Hazard ratios (95% CI) for disease transitions from baseline (CMD-free) to FCMD and CMM by quartiles of the baseline Elderly Dietary Index (EDI) in BRHS participants aged 60–79 years in 1998–2000 (*n* = 2873)

Baseline EDI quartiles	First cardiometabolic disease (*N* = 891) from baseline		Cardiometabolic multimorbidity (*N* = 109) from FCMD	
	No. of events	HR (95% CI)	No. of events	HR (95% CI)
Q1 (EDI score 9–22, *n* = 793)	264	Ref	33	Ref
Q2 (EDI score 23–24, *n* = 749)	238	0.97 (0.81, 1.16)	25	0.59 (0.34, 1.02)
Q3 (EDI score 25–26, *n* = 651)	196	0.93 (0.77, 1.13)	26	0.86 (0.49, 1.48)
Q4 (EDI score 27–35, *n* = 680)	193	0.87 (0.72, 1.07)	25	0.80 (0.45, 1.42)

Model adjusted for age, BMI, waist circumference, smoking status, alcohol intake, physical activity, social class, National IMD, energy intake, and use of any lipid-lowering drugs

*FCMD* First cardiometabolic disease, *CMM* Cardiometabolic multimorbidity

*P < 0.05

The multi-state model results of fish/seafood consumption with CMM disease transitions are presented in Tables 4 and 5. The impact of fish/seafood intake was significantly stronger in the transition from FCMD to CMM compared to the transition from CMD-free to FCMD. The fully adjusted HRs (95% CI) for the transition of CMM from FCMD were 0.43 (0.23, 0.77) and 0.41 (0.24, 0.70) for consuming fish/seafood $\ge$ 3 days/week and 1–2 days/week, respectively, comparing to consuming fish/seafood < 1 day/week. Specifically, the significant impact of fish/seafood consumption was in the transition of CMM from first MI or T2D (Table 5). Though the association of fish/seafood intake with combined FCMD was not significant, higher intake of fish/seafood ($\ge$ 3 days/week) was found to be significantly associated with lower risk of first MI (HR: 0.68, 95% CI 0. 48, 0.97) (Table 5). In addition, consuming fish/seafood more frequent was related to a lower risk of death from baseline, but a higher risk of death from first stroke. During the follow-up period, given average or reference levels of covariates, the predicted percentages of participants who developed CMM (survived or died after that) was shown to be consistently smaller for participants consuming fish/seafood more than 1 day/week comparing to < 1 day/week (Online Resource Fig. 4).

**Table 4:** Hazard ratios (95% CI) for disease transitions from baseline (CMD-free) to FCMD and CMM by fish/seafood consumption in BRHS participants aged 60–79 years in 1998–2000 (*n* = 2873)

Baseline fish/seafood consumption	First cardiometabolic disease (*N* = 891) from baseline		Cardiometabolic multimorbidity (*N* = 109) from FCMD	
	No. of events	HR (95% CI)	No. of events	HR (95% CI)
< 1 day/week (*n* = 308)	103	Ref	22	Ref
$\ge$ 3 days/week (*n* = 950)	274	0.81 (0.64, 1.02)	30	0.43 (0.23, 0.77)*
1–2 days/week (*n* = 1615)	514	0.93 (0.75, 1.16)	57	0.41 (0.24, 0.70)*

Model adjusted for age, BMI, waist circumference, smoking status, alcohol intake, physical activity, social class, National IMD, energy intake, use of any lipid-lowering drugs, and modified EDI score without fish/seafood intake

Baseline fish/seafood consumption categories are presented in the order of presumably the least healthy intake (< 1 day/week), the second healthy intake (≥ 3 days/week), and the healthiest intake (1–2 days/week), based on EDI scoring criteria

*FCMD* First cardiometabolic disease, *CMM* Cardiometabolic multimorbidity

*P < 0.05

**Table 5:** Hazard ratios (95% CI) for disease transitions from baseline (CMD-free) to first MI, stroke, or T2D, CMM, and death by baseline fish/seafood consumption in BRHS participants aged 60–79 years in 1998–2000 (*n* = 2873)

Disease transition	No. of events	Hazard ratios (95% CI) for each disease transition		
		Baseline fish/seafood consumption		
		< 1 day/week (*n* = 308)	$\ge$ 3 days/week (*n* = 950)	1–2 days/week (*n* = 1615)
Baseline to FCMD				
Baseline to MI	354	Ref	0.68 (0.48, 0.97)*	0.74 (0.54, 1.03)
Baseline to stroke	285	Ref	0.88 (0.56, 1.40)	1.10 (0.72, 1.67)
Baseline to T2D	252	Ref	1.07 (0.69, 1.67)	1.23 (0.81, 1.86)
FCMD to CMM				
MI to CMM	31	Ref	0.40 (0.12, 1.36)	0.24 (0.08, 0.73)*
Stroke to CMM	33	Ref	0.63 (0.16, 2.48)	0.67 (0.21, 2.11)
T2D to CMM	45	Ref	0.26 (0.08, 0.81)*	0.46 (0.18, 1.17)
Baseline to death	1022	Ref	0.81 (0.65, 1.01)	0.81 (0.67, 1.00)*
FCMD to death				
MI to death	254	Ref	1.40 (0.80, 2.45)	0.95 (0.59, 1.54)
Stroke to death	173	Ref	2.46 (1.22, 4.97)*	1.49 (0.78, 2.86)
T2D to death	73	Ref	0.91 (0.35, 2.40)	1.41 (0.57, 3.48)
CMM to death	70	Ref	1.58 (0.62, 4.03)	0.53 (0.21, 1.30)

Model adjusted for age, BMI, waist circumference, smoking status, alcohol intake, physical activity, social class, National IMD, energy intake, use of any lipid-lowering drugs, and modified EDI score without fish/seafood intake

Baseline fish/seafood consumption categories are presented in the order of presumably the least healthy intake (< 1 day/week), the second healthy intake (≥ 3 days/week), and the healthiest intake (1–2 days/week), based on EDI scoring criteria

*FCMD* First cardiometabolic disease, *CMM* Cardiometabolic multimorbidity

*P < 0.05

## Discussion

The present study investigated the prospective associations of an a priori diet quality score, EDI, and its components, with risk of CMM in older British men. Our results showed that baseline adherence to the EDI did not relate significantly to the development of CMM. However, consuming fish/seafood more than 1 day/week was significantly associated with lower risk of CMM. The impact of fish/seafood consumption on CMM development was found to be stronger in the transition of CMM from FCMD than from baseline to FCMD.

Two prior prospective studies conducted in British and Chinese populations examined the potential role that diet played in the development of CMM [3, 12]. Findings from the Whitehall II cohort of 8270 middle-aged British participants showed that a poor dietary habit, defined as < 1 serving/day of fruit and vegetable consumption, was significantly associated with the risk of FCMD from CMD-free at baseline [HR (95% CI) 1.09 (1.00, 1.18)], but not with the risk of CMM developed from FCMD [HR (95% CI) 1.14 (0.96, 1.36)] [3]. A Chinese cohort involving approximately half million participants also found that a less healthy dietary habit, defined as non-daily eating of vegetables, fruits, and eggs, and eating red meats daily or less than weekly, was significantly related to a higher risk of FCMD [HR (95% CI) 1.13, (1.08, 1.18)], but not to the development of CMM [HR (95% CI) 1.04 (0.94, 1.16] [12]. Nevertheless, Han et al. study showed that a less healthy dietary habit was significantly associated with higher risk of CMM when stroke was the FCMD [HR (95% CI) 1.25 (1.05, 1.49) and 1.84 (1.04, 3.25) for ischaemic stroke and haemorrhagic stroke, respectively] [12].

Our study brought prior work further using a Mediterranean diet-based dietary score EDI to measure the baseline diet quality in an older British men cohort. However, we did not observe significant associations between baseline EDI and the risk of FCMD or CMM. Lack of significance in the current study may mainly be due to the aging cohort and limited study power. The BRHS cohort used in our study consisted of participants who were all older than 60 years. While adherence to the Mediterranean diet was suggested to be associated with improvements in risk factors of cardiovascular diseases [15], associations of the risk factors such as blood pressure and cholesterol level with cardiovascular diseases were found to be weaker in older adults than in younger adults, potentially mainly because of physiological changes with aging [36–39]. In addition, higher absolute risks of cardiometabolic diseases and mortality in the elderly could potentially diminish the observed associations. Study power could be another possible reason for the insignificant results, as we had a relatively small number of CMM cases (*n* = 109) in our cohort. The full disease transition estimates in the current study revealed a significant inverse association between baseline EDI quartiles and risk of mortality transitioning from FCMD. However, unexpected higher risks of transitions from first MI to CMM and from CMM to mortality were found comparing baseline EDI Q3 to Q1 (Online Resource Tables 6, 7). The unexpected higher risks in the current study may be mainly because of selection bias, specifically index event bias and survival bias [40]. For example, the risk estimate of CMM from first MI was computed based on participants who experienced and survived the first MI. However, prior evidence has shown that an unhealthy diet was associated with higher risk of all-cause mortality and CVD mortality in older adults [17], which suggested that participants in lower EDI quartiles may not have experienced or survived the first MI. This then may lead to biased risk estimate of the EDI with the subsequent CMM following first MI. Overall, the impacts of diet quality on CMM and its disease transitions warrant further investigation with a more general population of wider age range and larger cohorts.

Regarding dietary components of the EDI, we found that eating fish/seafood more than 1 day/week was associated with lower risk of CMM after adjusting for all potential confounders. More importantly, our study found that baseline consumption of fish/seafood played a significantly greater beneficial role in the transition of CMM from FCMD than from CMD-free to FCMD, with about 60% reduction in the risk of CMM from FCMD if consuming fish/seafood more than 1 day/week. The current study is the first to reveal the role of fish/seafood consumption in the development of CMM. Results of fish/seafood intake in specific disease transitions suggested a significant inverse association of higher fish/seafood intake with risk of first MI, and with risk of CMM developed from the first MI or T2D (Table 5). The significant result of consuming fish/seafood $\ge$ 3 days/week with lower risk of first MI is in line with previous evidence reporting the preventive impact of eating fish/seafood on coronary heart diseases [10, 41]. The impacts of fish/seafood consumption, however, were shown to be insignificant in the relationships with the other two FCMDs in our study. A recent systematic review also reached insignificant conclusion in the relationship between fish consumption and stroke based on 4 prospective cohort studies conducted in Western countries [41]. In addition, the impacts of fish/seafood consumption on risk of T2D were suggested to have geographical variations, with insignificant associations found in European cohorts [11].

Our study observed a significant impact of fish/seafood consumption in the disease transition of CMM from FCMD. This finding suggested a stronger protective role that fish/seafood consumption may have in older populations after developing the FCMD, especially after the development of first MI or first T2D. There have been several previous studies showing the potential secondary prevention effect of fish/seafood intakes [42, 43]. A recent analysis including three cohorts of patients with prior vascular diseases found that consuming fish at least two servings/week was significantly related to a lower risk of another major cardiovascular diseases (HR: 0.84, 95% CI 0.73, 0.96) [42]. Another meta-analysis including 57,394 patients with diabetes showed a significant reduction in the risk of developing CHD (RR: 0.61, 95% CI 0.29, 0.93) comparing the highest to the lowest fish consumption [43]. Biologically, fish/seafood is particularly beneficial to cardiovascular disease because of its relatively higher levels of seafood-derived long-chain n–3 polyunsaturated fatty acids (n–3 PUFAs), which were shown to be significantly related to improvements in endothelial function and inflammation, and blood lipid profiles [44]. An unexpectedly higher risk of mortality was observed in the transition of death from first stroke with higher fish/seafood consumption (particularly $\ge$ 3 days/week). This may also be mainly due to selection bias/index event bias. Notably, the current study did not specify types of fish/seafood consumed or their cooking methods. Previous evidence showed that fish/seafood types and cooking methods could affect the cardiometabolic health impacts of fish/seafood significantly, with nonfried fish/seafood showing more consistent positive results [41, 45]. Overall, our significant beneficial result of consuming more fish/seafood on CMM is in line with American Heart Association (AHA)’s advisory recommendation of consuming nonfried seafood 1–2 days/week for potential cardiovascular benefits [44].

In the last several decades, prevalence of multimorbidity has been rapidly increasing mainly because of the aging population worldwide and decreased mortality from major chronic diseases [5, 6]. A growing number of studies have been focused on exploring behavioral risk factors of multimorbidity. For example, a recent study involving 348,290 British adults found that prudent dietary pattern and a few food components were associated with risk of multimorbidity [46]. However, definitions of multimorbidity varied a lot across studies. With inter-related physiological pathways, some sets of chronic conditions tend to cluster together. Cardiometabolic conditions included in the current study are leading causes of deaths globally, and more importantly, with their common underlying risk factors and inter-related biological pathways, the co-occurrence of them is one of the most common multimorbidity patterns [47]. A prior UK cohort showed that 6% of middle-aged British adults developed CMM during a mean follow-up of 23.7 years [3]. Our older British men cohort had a lower percentage of CMM occurrence (3%) compared to the younger British adults’ cohort, and this may be because our older men cohort had a high all-cause mortality rate and high fatality rates of the first MI or stroke event. Nevertheless, results of the current study provide more insights into this leading multimorbidity pattern and its relationships with dietary factors, particularly in the elderly. Findings of our study could potentially influence future preventive programs and guidelines, and hence have considerable public health benefits on the management of multimorbidity for older adults.

The current study has several strengths. First, this study used a prospective population-based cohort that followed participants for a long period of time. Second, we used an FFQ that had been validated against weighted food intakes in British populations [22, 23], and participants’ dietary intakes were comparable to the survey results of the National Diet and Nutrition Survey [48]. In addition, the internal validity of the study was improved by adjusting for a range of potential confounding variables, including both sociodemographic and lifestyle factors. However, findings of the current study need to be considered in light of several limitations. First, the dietary measure of the study is prone to measurement error as the diet quality scores were derived based on one-time dietary measure at baseline. This could bias our estimates since we did not know if participants changed their dietary habits during follow-up, especially after the diagnosis of the first or second cardiometabolic disease. Besides, due to design consideration, we only excluded patients with baseline prevalent MI, but did not identify people who had previously received a CABG or PCI. Patients with chronic coronary syndromes at baseline are at higher risk of developing another cardiometabolic disease and may have a less healthy dietary habit. Including these patients in the current study may increase the risk estimates seen in the unhealthy diet group. However, as we did not observe significant HRs between diet quality groups in the current analyses, impact of this potential bias on our findings is minimal. It is also possible that patients with prevalent chronic coronary conditions may have modified their dietary habit and adopted a healthier diet, and this may bias the model estimates towards the null in the current analyses. Third, we had a relatively small number of cases recorded during follow-up, which reduced our study power in the analyses of associations and disease transitions. Fourth, our current study used a Mediterranean diet-adapted dietary index EDI that was specifically developed for older populations to measure diet quality. The FFQ used in the study did not collect more detailed dietary information, such as serving sizes and sodium intakes, so we were unable to derive and explore other diet quality scores, such as the Alternative Healthy Eating Index (AHEI) or Dietary Approaches to Stop Hypertension (DASH), that were developed to apply to general populations based on different dietary guidelines. This limited the generalizability of our study results to general populations. In addition, our cohort only involved British men who were mostly from European ethnic origin, so we could not explore whether our results generalize to other ethnic groups or women. Finally, due to the study’s observational nature, we could not rule out the effects of possible residual confounding, such as the misclassification of self-reported lifestyle factors or other unmeasured confounding factors.

## Conclusion

Our study is the first to investigate the associations of a priori diet quality score and dietary components with the risk of cardiometabolic multimorbidity. We did not observe a significant association between baseline adherence to the Mediterranean-adapted diet quality score (EDI) and the risk of CMM. However, fish/seafood consumption, a dietary component of the EDI, was found to be significantly inversely related to the risk of CMM, particularly in the transition of CMM from FCMD.

To conclude, results of the current study keep with the AHA and traditional Mediterranean diet recommendations of consuming nonfried fish/seafood 1–2 days/week, and further the potential health benefits towards reducing the risk of CMM. Further work with larger cohorts and more general populations, other diet quality indices, and more frequent dietary measurements are needed to better understand the relationship of diet quality and dietary components with risk of CMM.

## Supplementary Information

Below is the link to the electronic supplementary material.

Supplementary file1 (DOCX 842 KB)

## Data Availability

Restrictions apply to the availability of the data. Data can be made available with the permission of BRHS. E-mail: l.lennon@ucl.ac.uk.

## Code availability

Available if required.
