# Supplementary material for: Prospective associations between diet quality, dietary components, and risk of cardiometabolic multimorbidity in older British men
Source: Eur J Nutr. 2023 Jun 19;62(7):2793–804. doi: 10.1007/s00394-023-03193-x (PMC10468910; doi:10.1007/s00394-023-03193-x)
Supplement: Supplementary file 1 — Supplementary file1 (DOCX 842 KB) [file 394_2023_3193_MOESM1_ESM.docx]

**Prospective associations between diet quality, dietary components, and risk of cardiometabolic multimorbidity in older British men**

**European Journal of Nutrition**

**Qiaoye Wang^1*^, Amand Floriaan Schmidt^2,3^, Lucy T. Lennon^1^, Olia Papacosta^1^, Peter H. Whincup^4^, and S. Goya Wannamethee^1^**

^1^ Department of Primary Care and Population Health, Institute of Epidemiology and Health Care, University College London, London NW3 2PF, UK

^2^ Department of Population Science and Experimental Medicine, Institute of Cardiovascular Science, University College London, London WC1E 6DD, UK

^3^ Department of Cardiology, Amsterdam Cardiovascular Sciences, Amsterdam University Medical Centre, University of Amsterdam, Amsterdam, The Netherlands

^4^ Population Health Research Institute, St George’s University of London, London SW17 0RE, UK

^*^ Corresponding author: Qiaoye Wang, email: [qiaoye.wang.21@ucl.ac.uk](mailto:qiaoye.wang.21@ucl.ac.uk)

**Supplementary Material**

**Table 1.**

Elderly Dietary Index (EDI) components and scoring criteria

| **EDI Scoring** | | | | |
| --- | --- | --- | --- | --- |
| **Component** | **Score = 1** | **Score = 2** | **Score = 3** | **Score = 4** |
| Fruits | <1 day/week | 1-2 days/week | 3-6 days/week | Daily |
| Vegetables | <1 day/week | 1-2 days/week | 3-6 days/week | Daily |
| Cereals | <1 day/week | 1-2 days/week | 3-6 days/week | Daily |
| Legumes | Never/rarely | <1 day/week | ≥3 days/week | 1-2 days/week |
| Meat | ≥3 days/week | Never/rarely | <1 day/week | 1-2 days/week |
| Fish/Seafood | Never/rarely | <1 day/week | ≥3 days/week | 1-2 days/week |
| Bread | None | White | White and whole grain | Whole grain |
| Olive oil ^a^ | Never/Rarely | Tertile 1 of intake | Tertile 2 of intake | Tertile 3 of intake |
| Dairy | Full-fat milk and full-fat cheese | Semi-skimmed milk and full-fat cheese / full-fat milk and low-fat cheese | Skimmed milk and full-fat cheese | Skimmed/Semi-skimmed milk and low-fat cheese |

^a^ Never/rarely consume olive oil is the lowest intake category. For participants who consume olive oil at least once a month, consumption “tertiles” were used to categorize participants into the upper three intake groups.

**Fig 1.**

Distribution of Elderly Dietary Index (EDI) scoring in British Regional Heart Study (BRHS) participants (1998-2000), n=2873.


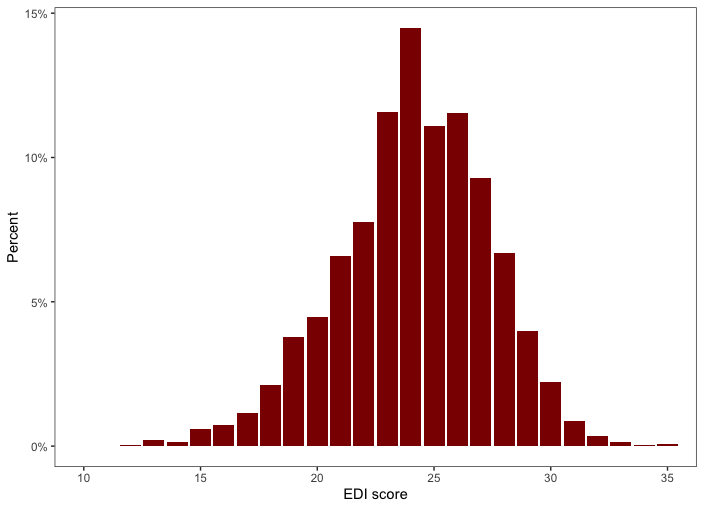


**Fig 2.**

Disease transition pattern from baseline (CMD-free) to first MI, first stroke, or first T2D, and to CMM and death.

**
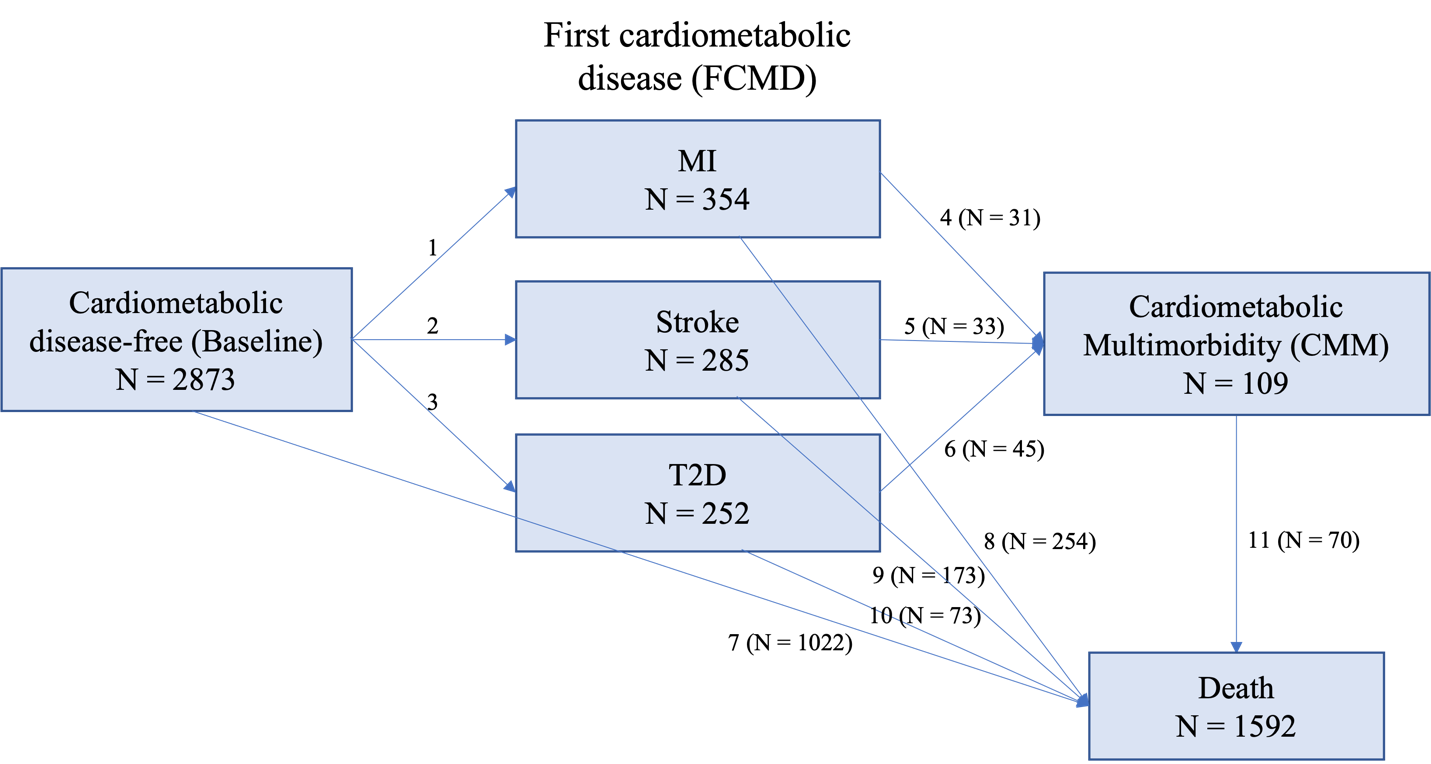
**

MI: Myocardial Infarction; T2D: Type 2 Diabetes

**Table 2.**

Comparison of complete cases and missing sample among BRHS participants free of prevalent cardiometabolic diseases at Q20 (n=3167).

| **Variable** | **Complete cases**  **(n = 2873)** | **Missing sample**  **(n = 294)** | **P value** |
| --- | --- | --- | --- |
| CMM, n (%) | 109 (3.8) | 10 (3.4) | 0.7 |
| Mean EDI score | 24.1 (3.3) | 23.5 (3.1), n = 62 | 0.1 |
| Age at baseline, years | 68.2 (5.5) | 70.2 (5.6), n = 294 | < 0.01 |
| Smoking status,  Current/recent smokers, % | 15.5 | N = 288  19.4 | < 0.01 |
| Alcohol intake  Heavy alcohol drinkers, % | 2.7 | N = 294  6.1 | < 0.01 |
| Physical activity,  Physically inactive, % | 8.6 | N = 294  11.9 | 0.02 |
| Social class,  Manual social class, % | 45.3 | N = 294  59.2 | < 0.01 |
| National IMD most deprived quintile, % | 14.7 | N = 291  26.8 | < 0.01 |
| BMI, kg/m^2^ | 26.7 (3.5) | 26.6 (3.9), n = 285 | 0.5 |
| Waist Circumference, cm | 96.4 (10.1) | 96.8 (10.5), n = 278 | 0.4 |
| Energy intake, kcal/day | 2147.9 (522.1) | 2012.3 (604.2), n = 173 | < 0.01 |
| Family history of diabetes, % | 11.2 | 8.2, n = 294 | 0.2 |
| Atrial Fibrilation based on ECG, % | 2.9 | 2.7, n = 293 | 0.8 |
| Use of any lipid-lowering drugs, % | 3.5 | 3.7 | 0.3 |
| Use of any blood pressure lowering drugs, % | 24.2 | 23.5 | 0.3 |
| Systolic blood pressure, mm Hg | 149.3 | 149.1, n = 293 | > 0.9 |
| Diastolic blood pressure, mm Hg | 85.8 (10.8) | 85.0 (12.0), n = 293 | 0.2 |
| Total cholesterol, mmol/L | 6.1 (1.1) | 5.9 (1.0), n = 279 | < 0.01 |
| Plasma HDL-C, mmol/L | 1.3 (0.3) | 1.4 (0.4), n = 277 | 0.3 |
| Plasma LDL cholesterol, mmol/L | 4.0 (1.0) | 3.8 (0.9), n = 276 | < 0.01 |
| Triglycerides, mmol/L | 1.8 (1.0) | 1.7 (1.2), n = 279 | 0.1 |

CMM, cardiometabolic multimorbidity; BMI, body mass index.

Values are presented as Mean (SD) or percentage unless stated otherwise.

Pearson’s chi-square test was used for all categorical variables.

Wilcoxon rank sum test was used for all continuous variables.

**Table 3.**

Prospective associations of baseline EDI dietary components with cardiometabolic multimorbidity in BRHS participants aged 60-79 years in 1998-2000 (n = 2873).

|  |  |  | **HRs (95% CI) of CMM** | | |
| --- | --- | --- | --- | --- | --- |
| **EDI Dietary Components** | **No. of events** | **Rate (per 1000 PY)** | **Model 1 ^a^** | **Model 2 ^b^** | **Model 3 ^c^** |
| **Fruits** |  |  |  |  |  |
| < 1 day/week (n = 244) | 8 | 1.64 | Ref | Ref | Ref |
| 1-2 days/week (n = 358) | 6 | 0.84 | 0.48 (0.17, 1.38) | 0.47 (0.16, 1.34) | 0.49 (0.17, 1.43) |
| 3-6 days/week (n = 1152) | 46 | 2.00 | 1.03 (0.49, 2.18) | 0.94 (0.44, 2.00) | 1.20 (0.55, 2.62) |
| Daily (n = 1119) | 49 | 2.19 | 1.06 (0.50, 2.24) | 1.00 (0.47, 2.12) | 1.39 (0.63, 3.09) |
| P for trend |  |  | 0.30 | 0.35 | 0.07 |
| **Vegetables** |  |  |  |  |  |
| < 1 day/week (n = 79) | 5 | 3.16 | Ref | Ref | Ref |
| 1-2 days/week (n = 265) | 11 | 2.08 | 0.60 (0.21, 1.73) | 0.53 (0.18, 1.52) | 0.52 (0.18, 1.54) |
| 3-6 days/week (n = 1723) | 60 | 1.74 | 0.47 (0.19, 1.16) | 0.44 (0.17, 1.08) | 0.46 (0.18, 1.19) |
| Daily (n = 806) | 33 | 2.05 | 0.51 (0.20, 1.29) | 0.49 (0.19, 1.26) | 0.55 (0.20, 1.50) |
| P for trend |  |  | 0.35 | 0.46 | 0.77 |
| **Cereals** |  |  |  |  |  |
| < 1 day/week (n = 204) | 9 | 2.21 | Ref | Ref | Ref |
| 1-2 days/week (n = 277) | 8 | 1.44 | 0.70 (0.27, 1.82) | 0.66 (0.25, 1.71) | 0.63 (0.24, 1.66) |
| 3-6 days/week (n = 508) | 27 | 2.66 | 1.10 (0.51, 2.33) | 1.07 (0.50, 2.28) | 1.11 (0.51, 2.41) |
| Daily (n = 1884) | 65 | 1.73 | 0.68 (0.34, 1.36) | 0.72 (0.36, 1.44) | 0.77 (0.37, 1.61) |
| P for trend |  |  | 0.19 | 0.34 | 0.57 |
| **Legumes** |  |  |  |  |  |
| Never/rarely (n = 324) | 14 | 2.16 | Ref | Ref | Ref |
| < 1 day/week (n = 297) | 15 | 2.53 | 1.11 (0.53, 2.30) | 1.19 (0.57, 2.48) | 1.30 (0.62, 2.74) |
| $\geq$ 3 days/week (n = 813) | 36 | 2.21 | 0.97 (0.52, 1.80) | 1.06 (0.57, 1.97) | 1.12 (0.59, 2.13) |
| 1-2 days/week (n = 1439) | 44 | 1.53 | 0.66 (0.36, 1.20) | 0.69 (0.38, 1.27) | 0.74 (0.40, 1.37) |
| P for trend |  |  | 0.06 | 0.07 | 0.10 |
| **Meat ^d^** |  |  |  |  |  |
| $\geq$ 3 days/week (n = 2642) | 100 | 1.89 | Ref | Ref | Ref |
| < 1 day/week (n = 86) | 4 | 2.33 | 1.11 (0.41, 3.01) | 1.23 (0.45, 3.33) | 1.08 (0.38, 3.03) |
| 1-2 days/week (n = 145) | 5 | 1.72 | 0.82 (0.34, 2.02) | 0.83 (0.34, 2.04) | 0.99 (0.39, 2.48) |
| P for trend |  |  | 0.74 | 0.80 | 0.98 |
| **Fish/Seafood ^e^** |  |  |  |  |  |
| < 1 day/week (n = 308) | 22 | 3.57 | Ref | Ref | Ref |
| $\geq$ 3 days/week (n = 950) | 30 | 1.58 | 0.37 (0.21, 0.63) * | 0.38 (0.22, 0.67) * | 0.37 (0.20, 0.66) * |
| 1-2 days/week (n = 1615) | 57 | 1.76 | 0.41 (0.25, 0.68) * | 0.44 (0.27, 0.72) * | 0.44 (0.26, 0.73) * |
| P for trend |  |  | 0.01 | 0.02 | 0.03 |
| **Bread ^f^** |  |  |  |  |  |
| None and white (n = 758) | 26 | 1.72 | Ref | Ref | Ref |
| White and whole grain (n = 1313) | 58 | 2.21 | 1.13 (0.71, 1.80) | 1.17 (0.74, 1.87) | 1.33 (0.82, 2.15) |
| Whole grain (n = 802) | 25 | 1.56 | 0.79 (0.46, 1.38) | 0.87 (0.50, 1.51) | 1.03 (0.58, 1.85) |
| P for trend |  |  | 0.39 | 0.61 | 0.91 |
| **Olive oil** |  |  |  |  |  |
| Never/Rarely (n = 2033) | 74 | 1.82 | Ref | Ref | Ref |
| Tertile 1 of intake  (n = 315) | 18 | 2.86 | 1.40 (0.83, 2.35) | 1.46 (0.87, 2.46) | 1.86 (1.08, 3.20) * |
| Tertile 2 of intake  (n = 258) | 10 | 1.94 | 0.99 (0.51, 1.92) | 1.08 (0.56, 2.10) | 1.32 (0.67, 2.61) |
| Tertile 3 of intake  (n = 267) | 7 | 1.31 | 0.59 (0.27, 1.28) | 0.57 (0.26, 1.24) | 0.66 (0.30, 1.44) |
| P for trend |  |  | 0.36 | 0.39 | 0.79 |
| **Dairy** |  |  |  |  |  |
| Full-fat milk and full-fat cheese (n = 669) | 22 | 1.64 | Ref | Ref | Ref |
| Semi-skimmed milk and full-fat cheese / full-fat milk and low-fat cheese  (n = 1572) | 57 | 1.81 | 1.08 (0.66, 1.77) | 1.06 (0.65, 1.74) | 1.18 (0.71, 1.97) |
| Skimmed milk and full-fat cheese (n = 281) | 17 | 3.02 | 1.85 (0.98, 3.51) | 1.88 (0.99, 3.56) | 2.29 (1.16, 4.49) * |
| Skimmed/Semi-skimmed milk and low-fat cheese  (n = 351) | 13 | 1.85 | 1.25 (0.63, 2.49) | 1.27 (0.64, 2.53) | 1.44 (0.70, 2.95) |
| P for trend |  |  | 0.20 | 0.18 | 0.10 |

CMM, cardiometabolic multimorbidity; EDI, Elderly dietary index.

^a^ Model 1: Adjusted for age.

^b^ Model 2: Adjusted for model 1 + BMI.

^c^ Model 3: Adjusted for model 2 + waist circumference, smoking status, alcohol intake, physical activity, social class, National IMD, energy intake, use of any lipid-lowering drugs, and modified EDI score without food group of interest.

^d^ Due to small case numbers, never/rarely and < 1 day/week were combined to < 1 day/week for meat intake.

^e^ Due to small case numbers, never/rarely and < 1 day/week were combined to < 1 day/week for fish/seafood intake

^f^ Due to small case numbers, none and white were combined to none/white for bread intake.

* p < 0.05

**Table 4.**

Prospective associations of EDI quartiles and fish/seafood consumption with cardiometabolic multimorbidity in BRHS participants aged 60-79 years in 1998-2000, excluding patients with prevalent heart failure at baseline (n = 2859)

| **Baseline EDI Quartiles** | **HRs (95% CI) of CMM** |
| --- | --- |
| Q1 (EDI Score 9-22) | Ref |
| Q2 (EDI Score 23-24) | 0.71 (0.42, 1.21) |
| Q3 (EDI Score 25-26) | 0.94 (0.55, 1.60) |
| Q4 (EDI Score 27-35) | 0.89 (0.51, 1.55) |
| **Baseline Fish/Seafood Consumption ^a^** |  |
| < 1 day/week | Ref |
| $\geq$ 3 days/week | 0.37 (0.21, 0.66) * |
| 1-2 days/week | 0.43 (0.25, 0.72) * |

CMM, cardiometabolic multimorbidity.

^a^ The two least adherence intake groups were combined (never/rarely and < 1 day/week) into < 1 day/week due to small case numbers in the “never/rarely” consumption group.

Model was adjusted for age, BMI, waist circumference, smoking status, alcohol intake, physical activity, social class, National IMD, energy intake, use of any lipid-lowering drugs. An additional covariate, modified EDI score without fish/seafood intake, was adjusted in the fish/seafood consumption model.

**Table 5.**

Prospective associations of EDI quartiles and fish/seafood consumption with cardiometabolic multimorbidity in BRHS participants aged 60-79 years in 1998-2000, after missing data imputation (n = 3167)

| **Baseline EDI Quartiles** | **HRs (95% CI) of CMM** |
| --- | --- |
| Q1 (EDI Score 9-22) | Ref |
| Q2 (EDI Score 23-24) | 0.72 (0.42, 1.21) |
| Q3 (EDI Score 25-26) | 0.91 (0.54, 1.54) |
| Q4 (EDI Score 27-35) | 0.90 (0.53, 1.54) |
| **Baseline Fish/Seafood Consumption ^a^** |  |
| < 1 day/week | Ref |
| $\geq$ 3 days/week | 0.39 (0.22, 0.70) * |
| 1-2 days/week | 0.50 (0.31, 0.83) * |

CMM, cardiometabolic multimorbidity.

^a^ The two least adherence intake groups were combined (never/rarely and < 1 day/week) into < 1 day/week due to small case numbers in the “never/rarely” consumption group.

Model was adjusted for age, BMI, waist circumference, smoking status, alcohol intake, physical activity, social class, National IMD, energy intake, use of any lipid-lowering drugs. An additional covariate, modified EDI score without fish/seafood intake, was adjusted in the fish/seafood consumption model.

**Table 6.**

Hazard Ratios (95% CI) for disease transitions from baseline (CMD-free) to FCMD, CMM, and death by quartiles of the Elderly Dietary Index (EDI) in BRHS participants aged 60-79 years in 1998-2000 (n = 2873)

|  |  | **Hazard Ratios (95% CI) for each disease transition** | | | |
| --- | --- | --- | --- | --- | --- |
|  |  | **Baseline EDI Quartiles** | | | |
| **Disease transition** | **No. of Events** | **Q1**  (EDI Score 9-22, n = 793) | **Q2**  (EDI score 23-24, n = 749) | **Q3**  (EDI score 25-26, n = 651) | **Q4**  (EDI score 27-35, n = 680) |
| Baseline to FCMD | 891 | Ref | 0.97 (0.81, 1.16) | 0.93 (0.77, 1.13) | 0.87 (0.72, 1.07) |
| FCMD to CMM | 109 | Ref | 0.59 (0.34, 1.02) | 0.86 (0.49, 1.48) | 0.80 (0.45, 1.42) |
| Baseline to death | 1022 | Ref | 0.90 (0.76, 1.07) | 0.93 (0.78, 1.12) | 0.84 (0.70, 1.02) |
| FCMD to death | 500 | Ref | 0.55 (0.43, 0.70) * | 0.68 (0.53, 0.88) * | 0.47 (0.35, 0.63) * |
| CMM to death | 70 | Ref | 2.45 (0.99, 6.06) | 4.40 (1.66, 11.66) * | 2.58 (0.94, 7.04) |

FCMD: First cardiometabolic disease; CMM: Cardiometabolic multimorbidity

Model adjusted for age, BMI, waist circumference, smoking status, alcohol intake, physical activity, social class, National IMD, energy intake, and use of any lipid-lowering drugs

* p < 0.05

**Table 7.**

Hazard Ratios (95% CI) for disease transitions from baseline (CMD-free) to first MI, Stroke, or T2D, CMM, and death by quartiles of the Elderly Dietary Index (EDI) in BRHS participants aged 60-79 years in 1998-2000 (n = 2873)

|  |  | **Hazard Ratios (95% CI) for each disease transition** | | | |
| --- | --- | --- | --- | --- | --- |
|  |  | **Baseline EDI Quartiles** | | | |
| **Disease transition** | **No. of Events** | **Q1**  (EDI Score 9-22, n = 793) | **Q2**  (EDI score 23-24, n = 749) | **Q3**  (EDI score 25-26, n = 651) | **Q4**  (EDI score 27-35, n = 680) |
| **Baseline to FCMD** |  |  |  |  |  |
| Baseline to MI | 354 | Ref | 1.04 (0.79, 1.38) | 0.96 (0.71, 1.30) | 0.75 (0.54, 1.04) |
| Baseline to Stroke | 285 | Ref | 0.90 (0.64, 1.25) | 1.07 (0.76, 1.50) | 1.01 (0.71, 1.43) |
| Baseline to T2D | 252 | Ref | 1.01 (0.72, 1.40) | 0.84 (0.57, 1.22) | 0.96 (0.67, 1.38) |
|  |  |  |  |  |  |
| **FCMD to CMM** |  |  |  |  |  |
| MI to CMM | 31 | Ref | 0.85 (0.23, 3.07) | 4.21 (1.05, 16.71) * | 3.45 (0.83, 14.34) |
| Stroke to CMM | 33 | Ref | 0.28 (0.07, 1.15) | 0.38 (0.12, 1.26) | 1.25 (0.39, 3.96) |
| T2D to CMM | 45 | Ref | 0.77 (0.35, 1.72) | 0.67 (0.28, 1.60) | 0.38 (0.13, 1.13) |
|  |  |  |  |  |  |
| **Baseline to death** | 1022 | Ref | 0.91 (0.77, 1.08) | 0.94 (0.79, 1.13) | 0.85 (0.71, 1.03) |
|  |  |  |  |  |  |
| **FCMD to death** |  |  |  |  |  |
| MI to death | 254 | Ref | 0.73 (0.49, 1.07) | 1.04 (0.67, 1.59) | 0.68 (0.43, 1.07) |
| Stroke to death | 173 | Ref | 0.41 (0.25, 0.67) * | 0.63 (0.39, 0.99) * | 0.52 (0.30, 0.91) * |
| T2D to death | 73 | Ref | 0.45 (0.22, 0.91) * | 0.69 (0.34, 1.37) | 0.41 (0.19, 0.92) * |
|  |  |  |  |  |  |
| **CMM to death** | 70 | Ref | 2.19 (0.88, 5.43) | 4.24(1.49, 12.05) * | 1.58 (0.58, 4.34) |

FCMD: First cardiometabolic disease; CMM: Cardiometabolic multimorbidity

Model adjusted for age, BMI, waist circumference, smoking status, alcohol intake, physical activity, social class, National IMD, energy intake, and use of any lipid-lowering drugs

* p < 0.05

**Fig 3.**

Disease transition probabilities from baseline to incident FCMD, CMM, and death with or without CMD and CMM for BRHS participants by baseline EDI quartiles.


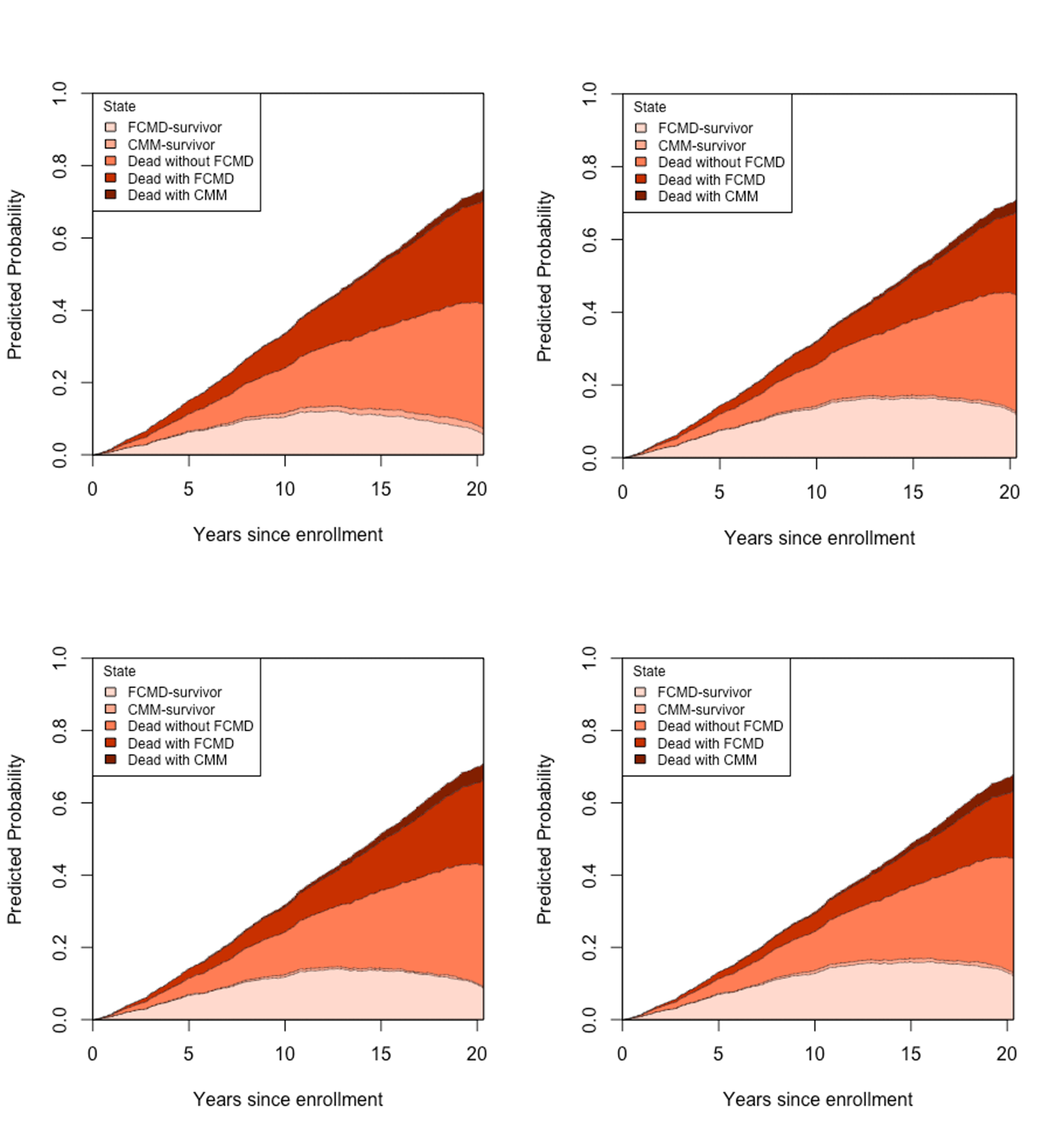


FCMD: First cardiometabolic disease; CMM: Cardiometabolic multimorbidity.

Disease transition probabilities were computed for participants with baseline EDI Q1 (top-left), baseline EDI Q2 (top-right), baseline EDI Q3 (bottom-left), and baseline EDI Q4 (bottom-right). All covariates were set to the average level or reference level of the BRHS population in the present analysis.

**Table 8.**

Hazard Ratios (95% CI) for disease transitions from baseline (CMD-free) to FCMD, CMM, and death by baseline fish/seafood consumption in BRHS participants aged 60-79 years in 1998-2000 (n = 2873)

|  |  | **Hazard Ratios (95% CI) for each disease transition** | | |
| --- | --- | --- | --- | --- |
|  |  | **Baseline Fish/Seafood Consumption** | | |
| **Disease transition** | **No. of Events** | < 1 day/week (n = 308) | $\geq$ 3 days/week (n = 950) | 1-2 days/week (n = 1615) |
| Baseline to FCMD | 891 | Ref | 0.81 (0.64, 1.02) | 0.93 (0.75, 1.16) |
| FCMD to CMM | 109 | Ref | 0.43 (0.23, 0.77) * | 0.41 (0.24, 0.70) * |
| Baseline to death | 1022 | Ref | 0.81 (0.65, 1.01) | 0.81 (0.67, 1.00) * |
| FCMD to death | 500 | Ref | 1.09 (0.77, 1.52) | 0.82 (0.60, 1.11) |
| CMM to death | 70 | Ref | 1.58 (0.62, 4.03) | 0.53 (0.21, 1.30) |

FCMD: First cardiometabolic disease; CMM: Cardiometabolic multimorbidity

Model adjusted for age, BMI, waist circumference, smoking status, alcohol intake, physical activity, social class, National IMD, energy intake, use of any lipid-lowering drugs, and modified EDI score without fish/seafood intake.

**Fig 4.**

Disease transition probabilities from baseline to incident FCMD, CMM, and death with or without CMD and CMM by baseline fish/seafood consumption frequencies


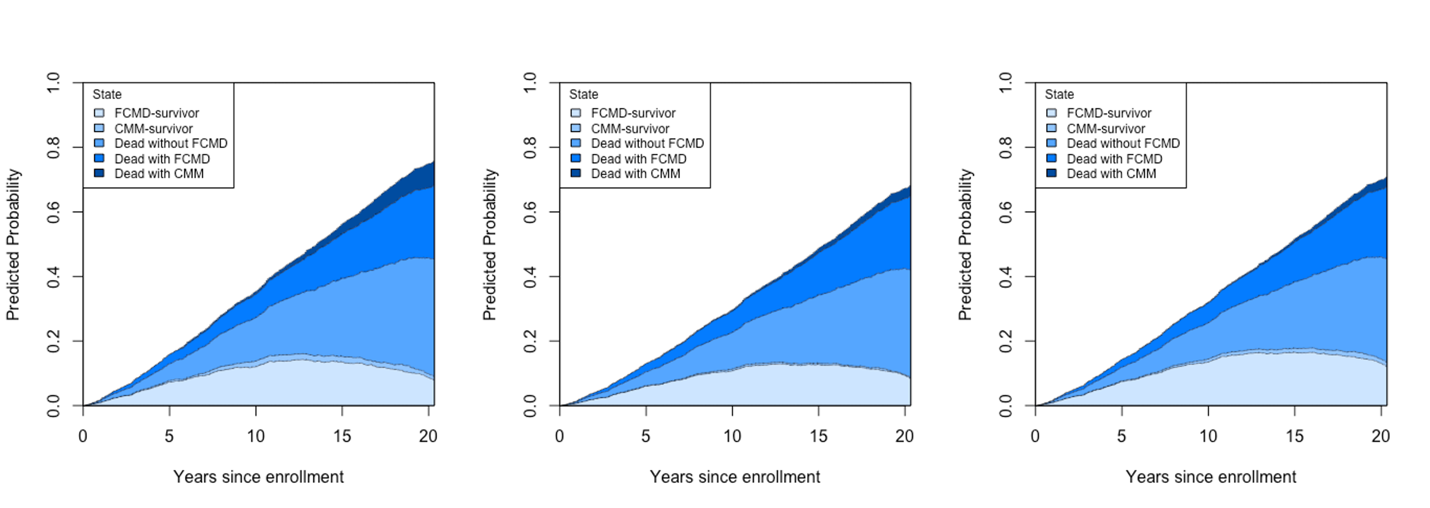


FCMD: First cardiometabolic disease; CMM: Cardiometabolic multimorbidity.

Disease transition probabilities were computed for participants with baseline fish/seafood consumption < 1 day/week (left), baseline fish/seafood consumption$\geq$3 days/week (middle), and baseline fish/seafood consumption 1-2 days/week (right). All covariates were set to the average level or reference level of the BRHS population in the present analysis.
